# Team performance in resuscitation teams: Comparison and critique of two recently developed scoring tools^☆^

**DOI:** 10.1016/j.resuscitation.2012.04.015

**Published:** 2012-12

**Authors:** Anthony McKay, Susanna T. Walker, Stephen J. Brett, Charles Vincent, Nick Sevdalis

**Affiliations:** a Department of Resuscitation and Outreach, St. Mary's Hospital, Imperial College Healthcare NHS Trust, Praed Street, London W2 1NY, UK; b Clinical Safety Research Unit, Department of Surgery and Cancer, Imperial College London, St. Mary's Hospital Campus, 10th Floor QEQM Building, St. Mary's Hospital, Praed Street, London W2 1NY, UK; c Center for Perioperative Medicine and Critical Care Research, Department of Anaesthesia and Intensive Care, Hammersmith Hospital, Imperial College Healthcare NHS Trust, Du Cane Road, London W12 0HS, UK

**Keywords:** Resuscitation teams, Non-technical skills, Teamworking skills, Adverse events, Patient safety, Assessment tools

## Abstract

**Background and aim:**

Following high profile errors resulting in patient harm and attracting negative publicity, the healthcare sector has begun to focus on training non-technical teamworking skills as one way of reducing the rate of adverse events. Within the area of resuscitation, two tools have been developed recently aiming to assess these skills – TEAM and OSCAR. The aims of the study reported here were:

1. To determine the inter-rater reliability of the tools in assessing performance within the context of resuscitation.
2. To correlate scores of the same resuscitation teams episodes using both tools, thereby determining their concurrent validity within the context of resuscitation.
3. To carry out a critique of both tools and establish how best each one may be utilised.

**Methods:**

The study consisted of two phases – reliability assessment; and content comparison, and correlation. Assessments were made by two resuscitation experts, who watched 24 pre-recorded resuscitation simulations, and independently rated team behaviours using both tools. The tools were critically appraised, and correlation between overall score surrogates was assessed.

**Results:**

Both OSCAR and TEAM achieved high levels of inter-rater reliability (in the form of adequate intra-class coefficients) and minor significant differences between Wilcoxon tests. Comparison of the scores from both tools demonstrated a high degree of correlation (and hence concurrent validity). Finally, critique of each tool highlighted differences in length and complexity.

**Conclusion:**

Both OSCAR and TEAM can be used to assess resuscitation teams in a simulated environment, with the tools correlating well with one another. We envisage a role for both tools – with TEAM giving a quick, global assessment of the team, but OSCAR enabling more detailed breakdown of the assessment, facilitating feedback, and identifying areas of weakness for future training.

## 1 Introduction

In many potentially high-risk industries, like commercial aviation, the nuclear industry, and the oil industry, analyses of human errors have consistently revealed that “human factors”, specifically teamworking skills, are often at the heart of errors and failures.^1–3^ To reduce human errors and promote safety and high reliability, assessment and training of a range of operators’ “non-technical” teamworking skills has been introduced in these industries (often termed “crew resource management” (CRM) training).^4,5^ Non-technical skills, including monitoring/situational awareness, decision-making, leadership, and communication skills,^6,7^ reflect how operators behave and think during routine activity, but also when crises occur and need to be safely managed.^8^

Following high profile errors resulting in patient harm and attracting negative publicity,^9,10^ the healthcare sector as a whole has also turned its attention to non-technical skills – with the specialties of anaesthesia and surgery paving the way. Within these specialties, CRM-styled training has been developed,^11,12^ and a range of tools that capture non-technical skills and assess team performance, typically via observation, have been developed and validated for use in real clinical settings as well as in simulation-based training environments.^13–16^

Non-technical skills are particularly relevant to resuscitation settings and acutely ill patients.^7,17^ When compared with the general hospital population, emergency patient care is especially susceptible to adverse events,^18,19^ and Ornato et al^20^ have demonstrated that these are associated with decreased survival of adults with in-hospital cardiac arrest. A variety of factors are thought to contribute to this, including time-pressured decision-making, an unstable patient population, an increased number of invasive procedures, and rapid assembly of ad hoc teams. This supports the need for non-technical skills awareness, and training for staff caring for these patients.^21,22^ Studies have also shown that effective teamwork may counteract problems with staffing and management, which in itself may reduce the incidence of adverse events.^23,24^

Specifically within the area of resuscitation, two different tools have been developed recently aiming to capture team performance and skills. The first one to be published in the literature was the Team Emergency Assessment Measure (TEAM) (Supplementary Online Appendix A), developed by an Australian research group.^25^ TEAM rates 11 behavioural aspects of the whole team on a Likert scale of 0–4, with an additional overall team score rated from 1 to 10. The behaviours that are measured are broken down into Leadership, Teamwork (including communication, co-operation and monitoring/situational awareness), and Task Management.

The second tool is the Observational Skill-based Clinical Assessment tool for Resuscitation (OSCAR) (Supplementary Online Appendix B), developed by our own research group.^26^ OSCAR was based on a rating tool previously developed and extensively validated for use in operating theatre settings, called the Observational Teamwork Assessment for Surgery (OTAS).^14,15^ OSCAR rates the performance of individual sub-teams within a standard resuscitation team (anaesthetists, physicians and nurses) across six teamwork-related behaviours (communication, co-operation, co-ordination, monitoring/situational awareness, leadership, and decision-making). Examples of “ideal” behaviours are given for each team member in each behaviour mode category to assist the assessor in determining ability. Each behaviour is rated on a 0–6 Likert scale with an additional “overall” score given for each section.

Another tool was developed almost concurrently by Andersen et al. in Denmark.^27^ However, this rates entire team behaviours on a dichotomous (“yes” and “no”) scale in a checklist format. The distinction between technical and non-technical performance within it is not as clear as it is within either OSCAR or TEAM. Due to these differences in skill content and coverage, we chose not to include this third tool in the direct comparison.

TEAM and OSCAR have been developed independently but are similar in their aim to capture team processes and performance. The aim of this study was to compare psychometrically the two tools, and determine the overall validity of the skills-assessment that is quantified within the context of resuscitation. Psychometric comparison in tools that involve observational assessment should include statistical evaluation of inter-rater reliability – in other words, the level of agreement between assessors using the tools. High reliability indicates that a tool produces consistent results across different assessors.^28^ Therefore the first two research questions that we addressed were:

What is the inter-rater reliability of OSCAR?

What is the inter-rater reliability of TEAM?

Moreover, given that OSCAR and TEAM aims to capture very similar skill sets, albeit in subtly different ways, we also directly compared assessments of the same resuscitation teams carried out using each one of the two tools. This is a question of concurrent validity, which addresses whether two instruments designed to assess similar skills and behaviours actually produce comparable assessments when used concurrently.^28^ Our final research question, therefore, was:

To what extent do OSCAR and TEAM scores correlate (i.e., statistically measure similar team characteristics)?

## 2 Methods

### 2.1 Procedure

#### 2.1.1 Phase 1 – reliability assessment

This phase aimed to assess the inter-rater reliability of both tools to ensure that they can be used reliably in assessing team skills in resuscitation contexts. Reliability assessment was performed by watching 24 pre-recorded resuscitation simulations (Supplementary Online Appendix C). The simulations had all been performed by cardiac arrest teams from our hospital (teaching hospital, London, UK). Twenty took place within the hospital's simulation centre, with small resuscitation teams consisting of a physician, an anaesthetist, and two nurses. These lasted an average of 5.5 min each. Four additional simulations were carried out “in situ” in clinical areas of the hospital, performed by the real on-duty resuscitation team for the day. These were inevitably longer simulations, and lasted an average of 13.5 min each.

The simulation recordings were watched by two resuscitation experts; one resuscitation officer (AMcK), and one anaesthetist (SW). Assessors were kept blinded to each other's ratings throughout this phase, and were trained in their observations prior to the beginning of the study. Each assessor watched each video once and applied both tools (i.e., OSCAR and TEAM).

#### 2.1.2 Phase 2 – content comparison and correlation of scorings

In this phase, the structure and use of the two tools were critically compared, and then the team ratings they generated were statistically correlated and plotted. Strong positive correlations between the two tools would provide evidence that they are broadly quantifying the same skill-sets (i.e., evidence for concurrent validity).

### 2.2 Statistical analyses

Data analyses were carried out using SPSS v.18.0 (SPSS Inc., Chicago, IL, USA). Inter-rater reliability refers to the level of agreement between two (or more) assessors using an assessment instrument. Intraclass correlation coefficients (ICC) were used to assess this in both TEAM and OSCAR, as recommended in the literature – with ICC values of 0.70 or higher indicating adequate agreement in scoring.^22^ Moreover, we also carried out non-parametric Wilcoxon tests to test whether the average scores allocated by each assessor were significantly different (non-significant results would indicate the desirable consistency in the scoring between the two assessors).^14^ Concurrent validity was assessed using non-parametric Spearman's rho correlation coefficients between OSCAR and TEAM scores. Scatterplots of these correlations, as well as Bland–Altman plots were produced. Bland–Altman plots are typically used to assess the level of agreement between two different measurement tools.^29^

Given the differences in the structure of the two tools, some algebraic manipulation was necessary for the correlational analyses to be possible. We computed an average score on each tool and expressed it as a percentage score (%). For the TEAM tool, we based the analyses on the first 11 questions, which are all scored on 0–4 point scales. The final question that assesses global performance on a 10-point scale was not included in this analysis, as it is scored on a different scale, it does not assess an individual skill or behaviour, and there is no OSCAR equivalent for comparison. TEAM scores, potentially ranging between 0 and 44, were then expressed as a percentage. For the OSCAR tool, there are six behaviours scored separately for three subgroups (anaesthetists, physicians, and nurses) – therefore a total of 18 ratings. Each rating is made on a 0–6 scale, and therefore OSCAR total scores, potentially ranging between 0 and 108, were again expressed as a percentage to allow direct comparison with TEAM. This manipulation enabled us to make the direct statistical comparison of scorings using the OSCAR and TEAM tools. It is important to point out that whilst this overall percentage was fairly straightforward to calculate for the TEAM tool as it is devised to measure overall team performance, for OSCAR this overall percentage score was an aggregate of overall scores for different team members, which acted as a surrogate for the overall score.

## 3 Results

### 3.1 Phase 1 – reliability assessment

A total of 85 healthcare providers were assessed in the study; 55 in the “in situ” simulations, given that these are attended by complete resuscitation teams, and 30 in the simulation centre simulations, with each of these participants performing two different simulations.

### 3.2 Inter-rater reliability of OSCAR

Scores from both assessors were compared across the three subgroups (anaesthetists, physicians, nurses) and six behaviours that OSCAR captures. Of the 18 scored behaviours, all achieved highly significant ICC results, 11 of which were very high with results ≥0.70 (Table 1).

Table 2 illustrates the median (range) scores given by both assessors for each team group and behaviour. Wilcoxon comparison between the two sets of data clearly demonstrates no significant difference between scorings, with the exception of four behaviour modes – “Monitoring/situational awareness”, “Decision-making”, and “Co-ordination” for nursing staff, and “Co-operation” for anaesthetists. Even in these four occasions, however, the actual scores given were similar, with identical medians (4) for Monitoring/situational awareness and Co-operation scores.

### 3.3 Inter-rater reliability of TEAM

Ratings allocated by the two assessors were compared across the 11 individual behaviours that TEAM captures, as well as the final global assessment that the tool generates. All achieved highly significant ICC results, with 7 of the total 12 comparisons achieving ICC results ≥0.70 (Table 3).

Table 3 also summarises the descriptive statistics (median and range) of the ratings allocated to each one of the TEAM items by both assessors. Statistical comparisons (Wilcoxon) of these ratings revealed that 6 of the 12 ratings achieved significance – thereby suggesting some overall disagreement between assessors. However, once again, these differences were small in absolute terms (identical medians in four behaviours; medians that differed by 0.50 in the remaining two behaviours) and never led to a difference in the direction of the overall opinion of the assessors (i.e., one rating the team as “neutral” and the other as “good”).

Taken together, these findings indicate that two independent and blinded assessors from different backgrounds can use both OSCAR and TEAM to capture a range of different behaviours across the resuscitation team successfully (with slightly better overall reliability for the OSCAR scoring).

### 3.4 Phase 2 – content comparison and correlation of scorings (concurrent validity)

**Fig. 1:**
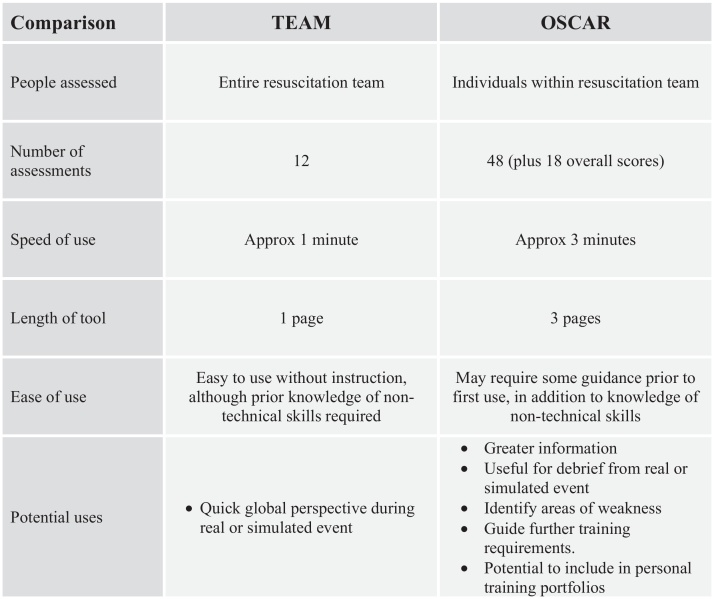
Chart comparing attributes of TEAM and OSCAR.

Fig. 1 provides a direct comparison of the two tools. The striking difference is that whilst OSCAR scores three sub-teams over six behaviour modes, with up to four behaviour examples given for each behaviour, the TEAM tool just rates the entire team over 12 different aspects. The result is that whilst OSCAR has a possible 48 individual scores and 18 overall scores to award, TEAM awards just 12 scores. This means, for example, that where there is one score for “Communication” in the TEAM tool, this facet is scored 10 times, with 3 additional “overall scores” in the OSCAR tool. OSCAR thus appears to be significantly more detailed in its skills coverage, though a longer tool to use.

**Fig. 2:**
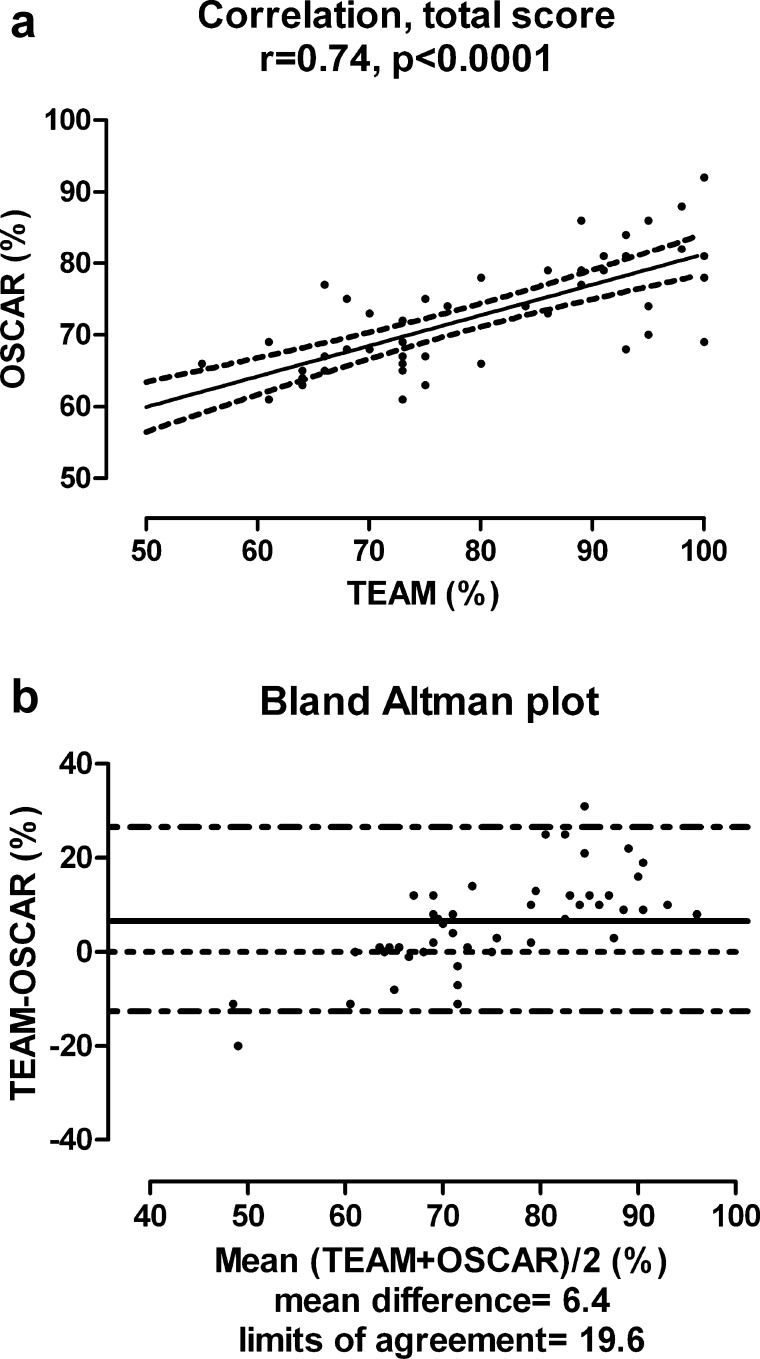
(a) Spearman's rho correlation and scatterplot between TEAM and OSCAR scores converted to percentages (%). (b) Bland–Altman plot of the TEAM and OSCAR percentage scores. The solid line represents the mean difference and the dashed lines 95% limits of agreement.

TEAM and OSCAR scores were converted to percentages (%) to allow direct statistical tool comparison. Overall, there was a strong correlation between TEAM and OSCAR scores (Spearmans's rho = 0.74, *p* < 0.0001; Fig. 2). The Bland–Altman plot demonstrates good agreement between the two tests, as shown by the relatively small number of points falling outside the 95% limits. Of note, however, is the fact that the tools had closest agreement at “average” scores. In general at low levels of performance the TEAM tool tended to score lower, and at higher levels of performance, the TEAM tool tended to score higher. This likely reflects the different scoring methods employed by the two tools.

## 4 Discussion

The aim of this study was to compare two recently published tools for the assessment of non-technical skills in the context of resuscitation – the Observational Skill-based Clinical Assessment tool for Resuscitation (OSCAR), and the Team Emergency Assessment Measure (TEAM). In summary, we have demonstrated strong inter-rater reliability for both tools when comparing ratings from two independent assessors, and strong correlation between the two tools when comparing percentage score surrogates. These results confirm that both tools are valid for use in the assessment of non-technical skills in resuscitation.

Whilst both tools achieved reasonable inter-rater reliability, findings were marginally better for the OSCAR tool than for the TEAM tool. We feel this is most likely to reflect the fact that the assessors had prior experience of using the OSCAR tool, having both used it during the initial development stages, and subsequent assessments of simulation teams, whereas this was the first time they had used the TEAM tool. One interpretation of this finding is that the TEAM tool is reasonably intuitive to use, such that assessors who are experienced and trained in assessing non-technical skills are able to use TEAM reasonably well even without prior tool-specific training. The lack of inter-assessor agreement in some elements of TEAM (compared to OSCAR), however, suggests that even adequately experienced assessors would likely benefit from tool-specific training. Training assessors prior to launching assessment programmes for team and non-technical skills is a recommendation that is increasingly emerging in the non-technical skills literature within healthcare.^30^

When we used the tools to rate resuscitation teams, we found that OSCAR requires a degree of concentration if unfamiliar with it, and takes a few minutes to complete thoroughly due to its length. However, if it is used properly, the result is a comprehensive assessment of individual team-members within the resuscitation team. For example, it was possible to identify that whilst nurses often lacked in their decision-making skills, they tended to score highly on co-operation skills, whereas the anaesthetic personnel tended to be strongest at demonstrating leadership qualities. We suspect this represents the traditional hierarchical structure and roles within healthcare, but may also reflect personalities of staff attracted to different specialties^31^; this is something that ought to be explored further in the future, using validated metrics of personality and self-perceptions alongside OSCAR. The more detailed information that stems from OSCAR, however, not only enables direct feedback to specific team members, but also enables the trainers to direct training to weaknesses within individual groups (e.g., helping nurses improve their decision-making skills). The assessment of individuals rather than an entire team also means that excellent participants and poorly performing candidates can be identified within the same team – thus tailoring feedback to individuals’ needs rather than allocating a single score to an entire team.

In contrast, the TEAM tool is shorter and quicker to use. The potential problem here, as mentioned above, is that by only giving a global score for the entire team it is not possible to identify weaker (or indeed excellent) team-members. Situations where a particularly strong team-member could inflate the scores of an entire team, even if other team members are well below average, can thus arise. This aspect of TEAM scoring, in our experience, occasionally made awarding a score challenging. Moreover, given that many teams have a mix of abilities, this may make TEAM assessors award many average scores, without adequate differentiation of levels of skills within the team. The higher agreement between the two tools at average levels of performance (compared to below or above average levels) is in accordance with this observation: TEAM scores may concentrate around the middle of the scale whereas with OSCAR some team members can indeed score higher than others – but when OSCAR scores are aggregated for comparison with TEAM, higher- and lower-scoring team members produce overall performance scores near the middle of the scale. Finally, scoring of a team's morale (TEAM Question 6) was problematic. Morale is a highly subjective concept, difficult to observe with any level of objectivity – and thus our assessors tended to allocate scores near the middle of the scale. This was highlighted as a problem by the original TEAM developers.^25^ Whilst team morale is extremely important, maybe this is something that should be discussed in a formal debrief, rather than rated using a tool – and in future research this item may be considered for removal. Overall, in our experience whilst the tool is useful as a start to assessing non-technical skills, it would not be possible to use this to identify specific training needs, and may not help identify poor performers.

In the light of this study, we envisage a role for both tools. TEAM is quick and easy to use, enabling fast global assessment of the team. OSCAR may have more potential as both an assessment, and also a training tool in giving far more detailed information about individual abilities. This enables it to be used as part of a formal structured debrief on courses, and training sessions.^32^ Having identified areas of weakness this can be used to inform and focus future training. We envisage that both tools can be used to assess level of performance in both simulated scenarios and real resuscitation episodes – non-technical behaviours can be linked to clinical tasks and immediate learning can take place both for individuals as well as for resuscitation teams as a whole.^33^ Doing so not just within pre-specified training episodes (i.e., simulation) but on-the-job directly embeds maintenance of high-level skills into daily clinical routine. Finally, there is also a possibility of individuals keeping their OSCAR assessments as part of their personal training portfolio.

### 4.1 Limitations

It is important to acknowledge that both tools have only been utilised in a simulated environment, although it is most likely this is where the greatest use will be for them in the near future. This may influence how the individuals and the teams behave and are subsequently rated, as the environment is “staged”. Evaluation of team performance in a simulated environment has its limitations for a number of reasons, such as length of scenario and attitudes towards simulation. It is difficult to fully appreciate how this study would translate into the rating of an actual cardiac arrest situation. Clinician performance may be more difficult to assess in real-time cardiac arrest situations, which may influence the reliability of both tools. Practically, although certainly desirable, a prospective evaluation of tools such as OSCAR and TEAM in real cardiac arrests is difficult because of the low incidence of such events – therefore further simulation-based evaluations are likely useful. Another limitation is the fact that using two scoring tools to rate the same simulation scenario may inevitably inflate the correlations between the tools (but it does not affect inter-assessor agreement). This is an inherent problem with any study where multiple assessments of various skills are carried out concurrently – and indeed of real life simulation and training settings, where the number of assessors/faculty is often limited. Further evaluation of correlation between the two tools reported here, and also other assessments are thus required – ideally with assessors scoring only one tool each.

## 5 Conclusion

We have demonstrated that two recently developed tools that assess teamworking and non-technical skills in resuscitation contexts, OSCAR and TEAM, can be used to assess reliably teamworking of cardiac arrest teams in a simulated environment. We have also shown that the two tools correlate reasonably well with one another, thereby providing evidence for the validity of their measurements. Taken together with the previous studies carried out for each one of these tools,^25,26^ the present findings corroborate the reliability and validity evidence base as well as practical feasibility of both tools. In the light of our findings, we propose that there is a place for both tools – with TEAM acting as a quick, instant assessment tool for the entire team, but OSCAR enabling more detailed breakdown of the assessment, facilitating constructive feedback to all team-members, and identifying areas of weakness in sub-teams or individuals that can help focus future directed learning.

## Funding

This research was funded by a grant from the Wellcome Trust, UK. The funding source of the study had no role in the study design, data collection, data analysis, data interpretation, writing of the report, or the decision to submit for publication. Walker, Vincent, and Sevdalis are affiliated with the Centre for Patient Safety and Service Quality at Imperial College Healthcare NHS Trust, which is funded by the UK's National Institute of Health Research. Brett wishes to acknowledge the support of the UK NIHR Comprehensive Biomedical Research Center Scheme.

## Conflict of interest statement

Brett is co-author on the worksheet “Quality of life after resuscitation” in the 2010 guideline revision. He has a research grant from Carefusion, and consults for Pfizer and Baxter Healthcare.

No other conflict of interest is declared.

## Ethics statement

Ethical approval was not required for this study, as it falls within the area of service evaluation and clinical audit.

## Figures and Tables

**Table 1:** OSCAR intraclass correlation coefficients between the two assessors across behaviours and subgroups (**p < 0.001).

Team subgroup	Behaviour/Skill					
	Communication	Co-operation	Co-ordination	Leadership	Monitoring	Decision-making
Anaesthetists	0.70**	0.61**	0.71**	0.68**	0.66**	0.61**
Physicians	0.85**	0.77**	0.87**	0.88**	0.79**	0.74**
Nurses	0.72**	0.68**	0.58**	0.75**	0.64**	0.75**

**Table 2:** OSCAR descriptive statistics (median/range) across behaviours, subgroups, and assessors; with significance of difference (*p*) between the two sets of ratings.

Behaviour/Skill	Anaesthetists			Physicians			Nurses		
	Assessor		Significance	Assessor		Significance	Assessor		Significance
	1	2		1	2		1	2	
Communication	4 (3–6)	4 (3–6)	0.53	5 (3–6)	5 (3–6)	1.00	4 (3–5)	4 (3–5)	0.48
Co-operation	4 (3–6)	4 (3–5)	0.03	5 (3–6)	5 (3–6)	0.71	4 (3–5)	4 (3–5)	0.48
Co-ordination	4 (3–5)	4 (3–5)	0.48	5 (3–6)	5 (3–6)	0.18	4.50 (3–6)	4 (3–5)	0.03
Leadership	4 (3–6)	4 (3–6)	0.11	5 (3–6)	5 (3–6)	0.18	4 (3–5)	4 (3–5)	0.16
Monitoring	4 (3–6)	4 (3–6)	0.76	5 (3–6)	5 (3–6)	0.26	4 (3–5)	4 (2–5)	0.01
Decision-making	5 (3–6)	4 (3–6)	0.25	5 (3–6)	5 (3–6)	0.32	4 (3–5)	3 (3–5)	0.005

Assessor 1 = anaesthetist, assessor 2 = resuscitation officer.

**Table 3:** TEAM intraclass correlation coefficients (**p < 0.001) and descriptive statistics (median /range) across items and assessors.

TEAM question	ICC	Assessor scores – median (range)		Significance (*p*)
		1	2	
1	0.59**	3.50 (1–4)	3 (1–4)	0.02
2	0.69**	3.50 (2–4)	3 (2–4)	0.03
3	0.70**	3 (1–4)	3 (1–4)	0.02
4	0.73**	3 (2–4)	3 (2–4)	0.71
5	0.78**	3 (2–4)	3 (2–4)	0.18
6	0.67**	3 (2–4)	2.50 (2–4)	0.06
7	0.73**	3 (2–4)	3 (1–4)	0.06
8	0.77**	3 (2–4)	3 (1–4)	0.03
9	0.54**	3 (1–4)	3 (1–4)	0.001
10	0.70**	3 (2–4)	3 (2–4)	0.41
11	0.63**	3 (2–4)	3 (2–4)	0.02
12	0.88**	7 (4–10)	7 (5–10)	0.21

Assessor 1 = anaesthetist, assessor 2 = resuscitation officer.
