# Supplementary material for: Team performance in resuscitation teams: Comparison and critique of two recently developed scoring tools
Source: Resuscitation. 2012 Dec;83(12):1478–83. doi: 10.1016/j.resuscitation.2012.04.015 (PMC3500685; doi:10.1016/j.resuscitation.2012.04.015)
Supplement: Supplementary file 1 [file mmc1.docx]

**Online Appendix A. TEAM tool.** (This tool was published in Resuscitation, Vol 81, Cooper S, Cant R, Porter J. “Rating medical emergency teamwork performance: development of the team emergency assessment measure (TEAM). Page 451, Copyright Elsevier (2010))

**
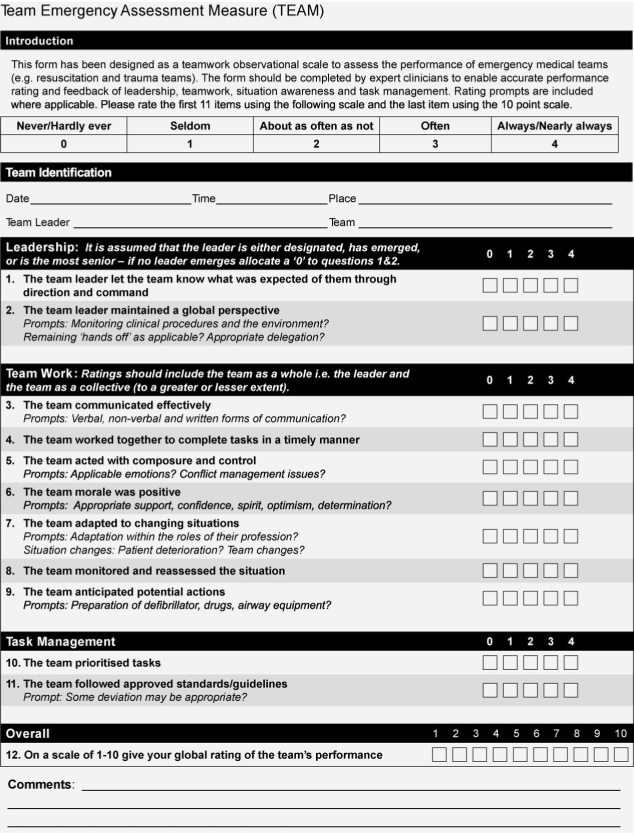
**

**Online Appendix B. The OSCAR tool**

**Observational Skill-based Clinical Assessment Tool for Resuscitation (OSCAR)**

**Date: Assessor: Candidate:**

0 = Team Severely Compromised 1 = Team Compromised

2 = Slight detriment to team 3 = Team neither enhanced or hindered

4 = Moderate enhancement to team 5 = High level of enhancement to team

6 = Highly effective in enhancing teamwork

**COMMUNICATION**

| **Anaesthetic Group (A)** | **Individual Behaviour Ratings** | | | | | | | **Global Behaviour Score (0-6)** |
| --- | --- | --- | --- | --- | --- | --- | --- | --- |
| Informs team whether patient is making respiratory effort | 0 | 1 | 2 | 3 | 4 | 5 | 6 |  |
| Informs team of any other relevant clinical signs eg dilated pupil, obvious injuries, signs of aspiration | 0 | 1 | 2 | 3 | 4 | 5 | 6 |  |
| Communication to team that they plan to intubate the patient if required | 0 | 1 | 2 | 3 | 4 | 5 | 6 |  |
| Requests patient history on arrival and communicates details to team, if required | 0 | 1 | 2 | 3 | 4 | 5 | 6 |  |
| **Physician Group (P)** | | | | | | | | |
| Reviews patient history and notes and communicates relevant details clearly to the team | 0 | 1 | 2 | 3 | 4 | 5 | 6 |  |
| Clear instructions communicated to the team regarding the arrest protocol | 0 | 1 | 2 | 3 | 4 | 5 | 6 |  |
| Encourages communication from sub-teams, and encourages team members to give opinions | 0 | 1 | 2 | 3 | 4 | 5 | 6 |  |
| **Nurse Group (N)** | | | | | | | | |
| Provides clear information about arrest events on arrival of arrest team | 0 | 1 | 2 | 3 | 4 | 5 | 6 |  |
| Senior nurse provides clear, audible requests to junior nurse when requesting equipment eg additional iv bags | 0 | 1 | 2 | 3 | 4 | 5 | 6 |  |
| Instructs other nurses on ward clearly how to assist with arrest or other ward duties as appropriate | 0 | 1 | 2 | 3 | 4 | 5 | 6 |  |

**CO-OPERATION**

| **Anaesthetic Group (A)** | **Individual Behaviour Ratings** | | | | | | | **Global Behaviour Score (0-6)** |
| --- | --- | --- | --- | --- | --- | --- | --- | --- |
| A-group provides information on request from P-group (eg about the airway) | 0 | 1 | 2 | 3 | 4 | 5 | 6 |  |
| A-group assists P-group in decision making in difficult scenarios | 0 | 1 | 2 | 3 | 4 | 5 | 6 |  |
| **Physician Group (P)** | | | | | | | | |
| Responds to questions from other team members about decisions made regarding the arrest | 0 | 1 | 2 | 3 | 4 | 5 | 6 |  |
| Supports less experienced members of P-group, and compensates for their lack of experience | 0 | 1 | 2 | 3 | 4 | 5 | 6 |  |
| **Nurse Group (N)** | | | | | | | | |
| Provide support and assistance to A-group and P-group when needed eg finding airway adjuncts | 0 | 1 | 2 | 3 | 4 | 5 | 6 |  |
| Help P-group locate items not routinely stocked on trolley, or missing from the trolley | 0 | 1 | 2 | 3 | 4 | 5 | 6 |  |
| Assist P-group with extra tasks eg sending bloods, contacting family, contacting labs etc | 0 | 1 | 2 | 3 | 4 | 5 | 6 |  |

**CO-ORDINATION**

| **Anaesthetic Group (A)** | **Individual Behaviour Ratings** | | | | | | | **Global Behaviour Score (0-6)** |
| --- | --- | --- | --- | --- | --- | --- | --- | --- |
| Information provided about changes in patient condition as they occur | 0 | 1 | 2 | 3 | 4 | 5 | 6 |  |
| A-group co-ordinate team to move patient eg floor to bed, up bed | 0 | 1 | 2 | 3 | 4 | 5 | 6 |  |
| **Physician Group (P)** | | | | | | | | |
| Notifies N and A groups of anticipated further requirements for patient resuscitation | 0 | 1 | 2 | 3 | 4 | 5 | 6 |  |
| Within P group, co-ordinates tasks such as taking of bloods, sending samples, sending ABG etc | 0 | 1 | 2 | 3 | 4 | 5 | 6 |  |
| **Nurse Group (N)** | | | | | | | | |
| Prepare Resus Trolley for use by team by bringing to bedside, turning monitor on etc | 0 | 1 | 2 | 3 | 4 | 5 | 6 |  |
| Prepare further drugs in readiness for their next required use eg prepare next adrenaline minijet | 0 | 1 | 2 | 3 | 4 | 5 | 6 |  |
| A Senior Nurse (Sister) is always present to provide backup to Staff Nurse | 0 | 1 | 2 | 3 | 4 | 5 | 6 |  |

**LEADERSHIP**

| **Anaesthetic Group (A)** | **Individual Behaviour Ratings** | | | | | | | **Global Behaviour Score (0-6)** |
| --- | --- | --- | --- | --- | --- | --- | --- | --- |
| Advises team on best management, and contingency plans for patient, and takes lead if required | 0 | 1 | 2 | 3 | 4 | 5 | 6 |  |
| Anaesthetist assertively takes a lead in Airway control and Ventilation on arrival at arrest | 0 | 1 | 2 | 3 | 4 | 5 | 6 |  |
| Lead Anaesthetist supervises and supports staff lacking familiarity with tasks or equipment | 0 | 1 | 2 | 3 | 4 | 5 | 6 |  |
| **Physician Group (P)** | | | | | | | | |
| Takes a lead and clearly instructs assistants with requirements for arrest and/or defers leadership as required if appropriate | 0 | 1 | 2 | 3 | 4 | 5 | 6 |  |
| Supervision given to staff lacking experience or familiarity with tasks or equipment | 0 | 1 | 2 | 3 | 4 | 5 | 6 |  |
| Instructs N-group of additional requirements eg recent blood results from computer, to call the family | 0 | 1 | 2 | 3 | 4 | 5 | 6 |  |
| **Nurse Group (N)** | | | | | | | | |
| Takes a lead with initial Basic Life Support attempts until Arrest Team arrive | 0 | 1 | 2 | 3 | 4 | 5 | 6 |  |
| Supervision and support given to junior or inexperienced members of N-team | 0 | 1 | 2 | 3 | 4 | 5 | 6 |  |

**MONITORING**

| **Anaesthetic Group (A)** | **Individual Behaviour Ratings** | | | | | | | **Global Behaviour Score (0-6)** |
| --- | --- | --- | --- | --- | --- | --- | --- | --- |
| Maintains monitoring of patient condition, signs of respiration, other clinical signs | 0 | 1 | 2 | 3 | 4 | 5 | 6 |  |
| Checks ventilation is adequate with regular blood gas analysis and amends ventilation accordingly | 0 | 1 | 2 | 3 | 4 | 5 | 6 |  |
| Confirms drug identity by checking syringe labeling prior to drug administration | 0 | 1 | 2 | 3 | 4 | 5 | 6 |  |
| **Physician Group (P)** | | | | | | | | |
| Maintains awareness of activities of other teams eg anaesthetist intubating | 0 | 1 | 2 | 3 | 4 | 5 | 6 |  |
| Monitors progress of resuscitation protocol with careful checking of time, and constant reassessment of limb of protocol and “extra considerations” | 0 | 1 | 2 | 3 | 4 | 5 | 6 |  |
| Checks team condition eg monitors for fatigue in team members from CPR and suggests team members change roles, take turns etc | 0 | 1 | 2 | 3 | 4 | 5 | 6 |  |
| **Nurse Group (N)** | | | | | | | | |
| Monitors patient dignity and considers well-being of other patients nearby | 0 | 1 | 2 | 3 | 4 | 5 | 6 |  |
| Maintains awareness of the needs of P and A groups | 0 | 1 | 2 | 3 | 4 | 5 | 6 |  |

**DECISION MAKING**

| **Anaesthetic Group (A)** | **Individual Behaviour Ratings** | | | | | | | **Global Behaviour Score (0-6)** |
| --- | --- | --- | --- | --- | --- | --- | --- | --- |
| Prompt identification of the problem | 0 | 1 | 2 | 3 | 4 | 5 | 6 |  |
| Rapidly and clearly outlines a strategy or plan, and asks for equipment | 0 | 1 | 2 | 3 | 4 | 5 | 6 |  |
| Anticipates potential problems and prepares accordingly – eg asks for further blood to be cross-matched | 0 | 1 | 2 | 3 | 4 | 5 | 6 |  |
| **Physician Group (P)** | | | | | | | | |
| Rapidly decides an appropriate course of action for continued resuscitation | 0 | 1 | 2 | 3 | 4 | 5 | 6 |  |
| Uses the team as a whole to help develop options – asks for opinions and processes them decisively | 0 | 1 | 2 | 3 | 4 | 5 | 6 |  |
| **Nurse Group (N)** | | | | | | | | |
| Prompt decision making during initial resuscitation attempts | 0 | 1 | 2 | 3 | 4 | 5 | 6 |  |
| Anticipates potential problems A and P teams may encounter eg pulls bed out from wall, clears area etc | 0 | 1 | 2 | 3 | 4 | 5 | 6 |  |
| Appropriate decision making regarding timing of initial decision to put out a cardiac arrest call | 0 | 1 | 2 | 3 | 4 | 5 | 6 |  |

**Online Appendix C – Simulation Scenarios**

In Situ Simulation Scenarios:

1. The “Patient” was in the side room of the acute admissions ward. Past medical history of alcoholic liver disease. Found by nursing staff performing routine observations to be unwell with a low blood pressure, tachycardia, and a large amount of malaena on the sheets. The nurse was asked to proceed as they would normally. The patient deteriorated further and had a PEA arrest.

2. The “Patient” was in the recovery bay in radiology having just had an endovascular aortic aneurysm repair. The nurse looking after the patient became concerned, and put out a medical emergency call because his observations suddenly deteriorated. She was concerned about rupture and internal bleeding. The patient proceeded to have a PEA arrest.

3. The “Patient” was the relative of a patient. He was feeling unwell with chest pain, and collapsed in the corridor outside the ward. A nurse was passing by and put out a cardiac arrest call. The patient had suffered a VF arrest secondary to Ischaemic Heart Disease

4. The “Patient” was in an empty bay in the Clinical Decision Unit, having presented the night before with a bad chest infection. A nurse from the ward was asked to review the patient as the Health Care Assistant noticed he didn’t look very well. He was struggling to breathe, had low oxygen saturations, and was showing signs of sepsis with a tachycardia and hypotension. The patient proceeded to a hypoxic and hypotensive PEA arrest.

Simulation Centre Scenarios

The simulation centre scenarios took place as part of another study. Small cardiac arrest teams were recruited – an anaesthetist, a medical registrar, and a nurse. They were asked to perform two scenarios, which were identical in each study session. For the purposes of this study, ten of these sessions were watched, therefore a total of twenty recordings.

Scenario 1:

“You have been called to the ward as part of the “Crash Team”. When the Health Care Assistant found Mr Jones in bed making groaning noises, having earlier complained of some abdominal pain, he immediately put out a cardiac arrest call. Mr Jones is currently an inpatient under the care of the Vascular team. He is known to have a large abdominal aortic aneurysm that the Vascular team is hoping to repair within the next few days”

Scenario 2:

“You have been called to the ward as part of the “Crash Team”. When the Health Care Assistant found Mr Smith in bed making groaning noises, having earlier complained of some central crushing chest pain, he immediately put out a cardiac arrest call. Mr Jones is currently an inpatient under the Cardiologists being investigated for crescendo angina. He has had a myocardial infarction in the past, although he can’t remember when it was. He currently takes medications for hypertension and hypercholesterolaemia, is known to be diabetic, and continues to smoke 40 cigarettes a day.”
